# Use of the Smart Excretion Care System Linked to Electronic Medical Records to Alleviate Nursing Burden and Enhance Patient Convenience: Mixed Methods Study

**DOI:** 10.2196/36324

**Published:** 2023-10-30

**Authors:** Hui-Woun Moon, Da Som Me Park, Se Young Jung

**Affiliations:** 1 Office of eHealth Research and Business Seoul National University Bundang Hospital Seongnam-si Republic of Korea; 2 Department of Family Medicine Seoul National University Bundang Hospital Seongnam-si Republic of Korea

**Keywords:** care, caregiving, instrument development, elderly, quality of life, ergonomics, focus groups, musculoskeletal, usability, feasibility, digital health intervention, digital health, health intervention, nursing, electronic medical record

## Abstract

**Background:**

The surge in older demographics has inevitably resulted in a heightened demand for health care, and a shortage of nursing staff is impending. Consequently, there is a growing demand for the development of nursing robots to assist patients with urinary and bowel elimination. However, no study has examined nurses’ opinions of smart devices that provide integrated nursing for patients’ urinary and bowel elimination needs.

**Objective:**

This study aimed to evaluate the feasibility of the Smart Excretion Care System tethered to electronic medical records in a tertiary hospital and community care setting and discuss the anticipated reductions in the burden of nursing care.

**Methods:**

Focus group interviews were conducted using the Consolidated Criteria for Reporting Qualitative Research (COREQ) guidelines. The interviews were conducted in March 2021 and involved 67 nurses who had worked at Seoul National University Bundang Hospital for more than 1 year and had experience in assisting patients with excretion care. Data were collected using purposive and snowball sampling methods.

**Results:**

A total of four themes relevant to the Smart Excretion Care System were found: (1) expected reductions in the burden of nursing care, (2) applicable indications (by departments and diseases), (3) preferred features/functions, and (4) expected benefits of using the Smart Excretion Care System in clinical facilities. Nurses from comprehensive nursing care wards had the highest burden when it came to excretion care. It was a common opinion that the Smart Excretion Care System would be very useful in intensive care units and should be applied first to patients with stroke or dementia.

**Conclusions:**

Excretion care is one of the most burdensome tasks for nurses, increasing their workload. The development of the Smart Excretion Care System as a digital health intervention could help improve nurses’ work efficiency, reduce their burden, and extend to caregivers and guardians.

## Introduction

The increasing age of the global population has placed a heightened demand on nursing care providers [1]. In particular, South Korea is witnessing a dramatic demographic shift, with an increasing proportion of the population aging [2,3]. Aging societies have demonstrated an increased need for basic nursing care that encompasses fundamental human needs [4]. These needs span various domains, such as respiration, nutrition, urinary and bowel elimination, hygiene, spiritual support, safety, counseling, and education [5-7]. Despite numerous studies on safety, nutrition, counseling, and education, there is a research gap in the domain of urinary and bowel elimination, with most studies merely conducting exploratory investigations or standardizing nursing practices [8,9].

Excretion care (EC) has emerged as a particularly pressing area of demand owing to the importance of dignified and appropriate assistance in maintaining patients’ comfort and dignity. However, this essential task can be physically demanding and time-consuming, escalating the need for innovation. Inefficient EC can also predispose patients to complications, such as urinary tract infections (UTIs) and pressure ulcers. Therefore, smart devices that can relieve the burden of EC and effectively communicate a patient’s excretory status to health care teams and caregivers are urgently needed.

Nursing care for urinary and bowel elimination entails activities related to excretion, such as clean intermittent catheterization, Foley catheterization, perineal care, and enemas [8,10]. The significance of caring for patients’ urinary and bowel elimination needs is evident in the considerable portion of nursing work devoted to this area. Previous studies indicate that between 49% and 84% of nursing cases report musculoskeletal disorders or back pain due to physically strenuous tasks, such as changing diapers [11-14]. Driven by a shortage of nursing staff and the physical toll of nursing work, there is an increasing demand for the development of nursing robots to aid in addressing the patients’ urinary and bowel elimination needs [15,16].

Currently, various smart devices, including smart mattresses [17-19], rehabilitation nursing robots [20,21], and patient transport robots [22,23] are being developed using the Internet of Things (IoT) or sensors. However, research on EC has predominantly focused on biological signal collection and fecal analysis using the Smart Excretion Care System [24,25]. No studies have been conducted on smart devices that provide comprehensive care for the urinary and bowel elimination requirements of patients.

Therefore, this study aimed to collect and examine nurses’ opinions of our Smart Excretion Care System. We demonstrated a prototype of a smart EC device developed for nurses seeking their views on the acceptability and usability of the product. The insights gained from their feedback provide invaluable guidance for researchers intending to develop similar systems in the future.

## Methods

### Setting

The research setting was a tertiary health care institution in South Korea equipped with 1335 beds. Patient care areas in the hospital are stratified into general wards (GWs), comprehensive nursing care wards (CNCWs), and intensive care units (ICUs), depending on the level of nursing care and patient severity. Typically, a nurse in a GW is responsible for overseeing 16 patients with an IQR between 12 and 24 years. Basic nursing tasks in GWs, including EC, bathing, and feeding, are facilitated predominantly by family members or caregivers. Regarding EC, nursing duties primarily involve changing diapers and documenting intake and output quantities. The national introduction of CNCWs in 2016 was intended to reduce the financial burden of public nursing care and enhance the quality of inpatient services [26]. CNCWs deliver 24-hour comprehensive patient care administered by professionally trained nurses without the support of informal caregivers, a model also employed in ICUs. All essential nursing care in both CNCWs and ICUs, including EC, is provided by trained nurses. However, the patient-to-nurse ratio in CNCWs, usually 5 or 6 to 1, is higher than in ICUs, where it is typically 2 or 3 to 1.

### Design

Using a mixed methods approach, we conducted both qualitative and quantitative analyses to capture a comprehensive understanding of the participants’ perspectives on the Smart Excretion Care System.

The quantitative analysis aimed to gather scaled feedback on potential enhancements and additions to the functions and features of the Smart Excretion Care System. In addition, this analysis identified patterns and tendencies in user preferences and expectations. An introductory survey was conducted to ensure that all the participants were adequately informed and ready to provide insights during the focus group interviews (Table S1 of Multimedia Appendix 1). The questionnaire was separated into 4 distinct categories: the nurses’ role in managing patient elimination, applicable indications, needs, and evaluation.

For the qualitative analysis, we implemented an exploratory content analysis of the transcribed interviews to comprehend the participants’ perspectives on the Smart Excretion Care System. The transcripts were rigorously examined and verified by the research team. The coauthors collaboratively generated, compared, reviewed, and validated codes and subthemes to ensure the reliability of the data. The study followed the Consolidated Criteria for Reporting Qualitative Research (COREQ) guidelines [27].

### Participants

We enrolled registered nurses from Seoul National University Bundang Hospital (SNUBH) who had a minimum of 1 year of professional experience. We engaged nurses from all departments at SNUBH without exclusion, except for career experience, and garnered diverse opinions and insights regarding their work environments. However, priority was given to applicants from ICU wards given their greater involvement in EC. SNUBH’s extensive history since its inception in 2003 of adopting various smart patient care technologies, such as smart bedside stations and a patient guide system, made its nursing staff an ideal cohort for this study [10,28-31]. For participant recruitment, we utilized purposive and snowball sampling, 2 nonprobability sampling methods that allow for strategic participant selection and expansion of potential study subjects through existing participant referrals, respectively [32,33]. Consistent with a previous study, our interview design included at least 5 participants per group [27].

To ascertain participants’ needs concerning medical devices that could assist them in managing patients’ EC, we introduced the nurses to the Smart Excretion Care System.

### Prototype of the Smart Excretion Care System

**Figure 1:**
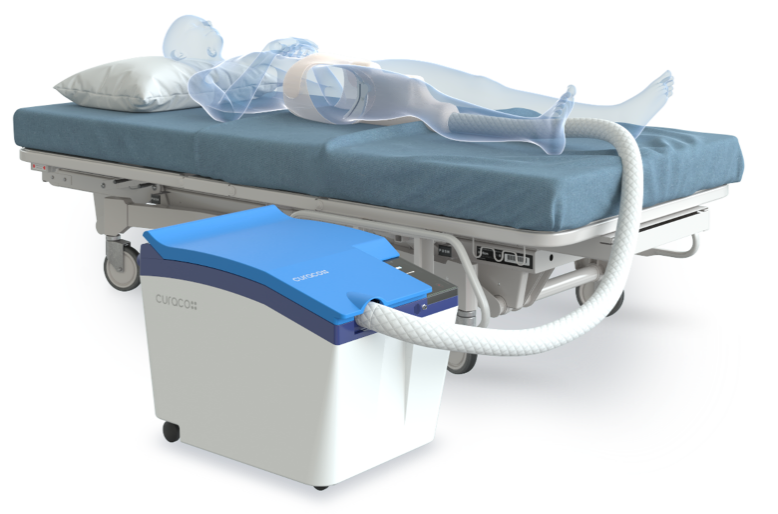
Prototype picture of the Smart Excretion Care System.

This prototype device was primarily developed to replace conventional diaper usage and to effectively manage patients’ excretions (Figure 1).

**Figure 2:**
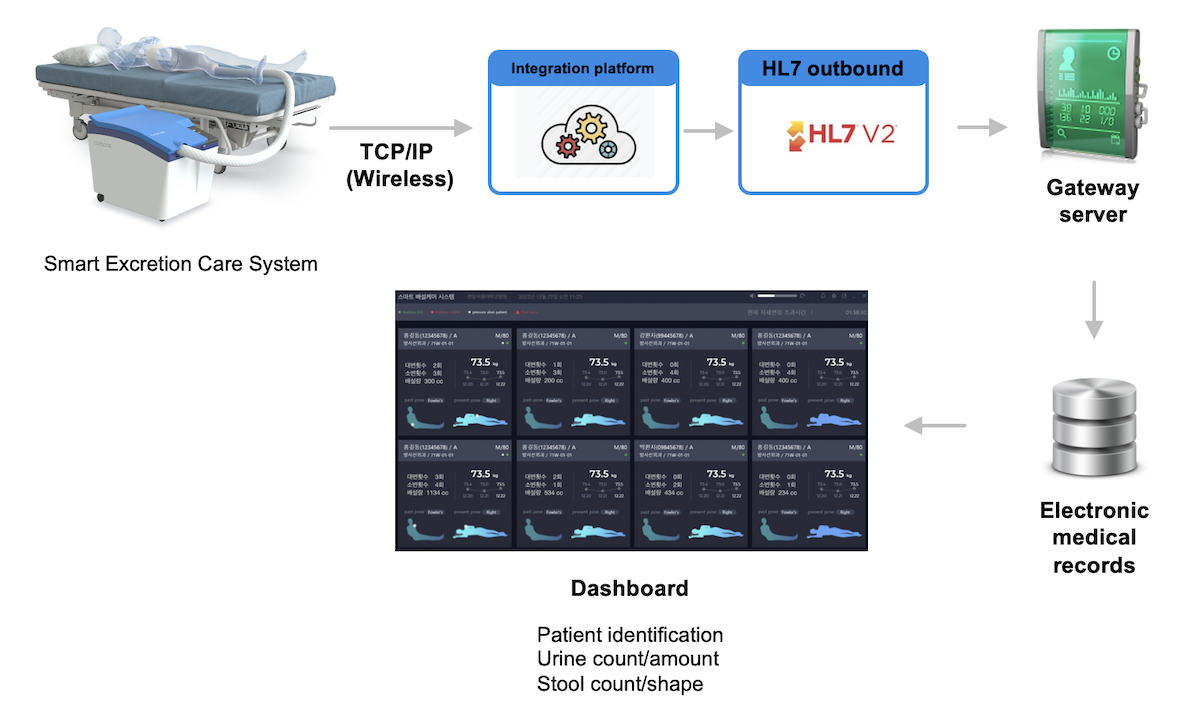
Integration of Smart Excretion Care System information with an electronic medical record (EMR) system. TCP/IP: Transmission Control Protocol/Internet Protocol.

It also facilitates the acquisition of digestive health data and prevents patients who are bedridden from developing skin complications resulting from urinary and fecal waste, such as pressure ulcers and incontinence-associated dermatitis (IAD). The prototype was engineered for individuals with mobility restrictions, such as individuals who are bedridden, disabled, or those who are older adults. The system employs advanced sensing technology to detect and remove urine and feces while simultaneously offering washing and air-drying features for enhanced hygiene and comfort, thus presenting an efficient substitute for traditional paper diapers. It ensures comprehensive washing, both anteriorly and posteriorly, accompanied by a warm air-drying mechanism and a deodorization feature, promising a hygienic user experience. It also includes a movable nozzle and sex-specific diaper cup, permitting 30-degree positional adjustments to boost cleaning efficiency and user comfort. The Smart Excretion Care System was also designed to seamlessly integrate patient data into electronic medical records (EMRs) (Figure 2).

The system communicates with a local server that collects and processes data from the care apparatus. The local server is linked to a central server capable of amalgamating and managing data from multiple local servers. The central server can also be integrated with an EMR system. This setup enables health care providers to monitor and track the health and hygiene status of individual users by incorporating crucial data into their comprehensive medical records. Moreover, this integration facilitates remote patient monitoring and personalized care.

### Data Collection

Data were collected through face-to-face interviews that began with structured questions but were designed to allow flexibility for open discussions. Conducted in groups of 6 (9%) to 8 (12%) participants grouped by ward, these sessions aimed to foster a candid exchange of views. While the interviews started with predetermined questions, the interviewer also posed additional, spontaneous questions based on the participants’ responses, making the process akin to a semistructured interview. Each session, lasting between 30 and 40 minutes, was recorded and transcribed with the participants’ consent.

### Data Analysis

#### Qualitative Analysis

For the qualitative data, we employed a thematic analysis approach to gain a deeper understanding of the underlying themes present in the nurses’ experiences and opinions regarding the Smart Excretion Care System. First, we conducted an exploratory content analysis of the transcribed interviews. The transcripts were read multiple times by different members of the research team to familiarize themselves with the data.

During the initial coding process, relevant portions of the text were identified and labeled. These codes were grouped according to their patterns and similarities, leading to potential themes. The emergent themes were reviewed and iteratively refined to ensure that they accurately represented the data set.

To enhance the reliability and validity of our findings, the transcripts were analyzed and cross-checked by multiple members of the research team. The generated codes and subthemes were compared, reviewed, and validated collaboratively by all coauthors. This collaborative approach was adopted to ensure the trustworthiness of the data and minimize potential biases.

#### Quantitative Analysis

Appropriate statistical analyses were conducted for the quantitative analysis. We aimed to obtain scaled results regarding the functions and features of the device that could be added or improved. This study also aimed to discern patterns and tendencies in the users’ preferences and expectations.

### Ethics Approval

The study was approved by the Institutional Review Board of Human Research of the SNUBH, Republic of Korea (B-2102-667-308). All participants were informed of their rights, including the option to withdraw from the study at any time.

## Results

### Overview

This study enrolled 67 participants, with no subsequent dropouts (Table 1). A significant majority of participants were female, and the predominant age group was between 20 and 39 years, with an equal number of participants in their 20s and 30s. Among the participants, 30 (45%) were drawn from GWs, 16 (24%) from CNCWs, 12 (18%) from ICUs, and 8 (12%) from the infection control team. In terms of career experience, 33 (49%) of the participants had between 3 and 5 years of experience, while 9 (14%) had 1 to 2 years of experience. Data saturation was achieved after interviewing approximately 45 (67%) of the participants.

**Table 1:** Demographic characteristics of the participants (N=67).

Characteristics			Values, n (%)
**Gender**			
	Male	2 (3)	
	Female	65 (97)	
**Age (years)**			
	20-29	29 (43)	
	30-39	29 (43)	
	40-49	8 (12)	
	50-59	1 (2)	
**Career (years)**			
	1-2	9 (14)	
	3-5	33 (49)	
	6-9	13 (19)	
	≥10	12 (18)	
**Department**			
	Intensive care unit	12 (18)	
	General ward	30 (45)	
	Comprehensive nursing care ward^a^	16 (24)	
	Infection control team	8 (12)	

^a^Refers to a ward where nurses solely undertake all aspects of patient care and visits from caregivers and family members are stringently limited.

### Quantitative Results

#### Preferences for Product Features

Based on the preliminary interviews and surveys, a comprehensive survey was conducted among the interviewees, who were actual users of the product, to gauge their preferences for product features and gather ranked responses regarding enhancements to be added to the current device (Table 2). A total of 67 respondents ranked each question on a 5-point Likert scale ranging from 1 (unnecessary) to 5 (necessary). The questionnaire was divided into 2 categories: product features and the functionality of information integration into an EMR. For product features, automatic flushing received the highest score, with an average of 4.7 points, while all other features averaged scores of 4.4 points or higher. Regarding the analysis functions, the ability to accurately measure the quantity of urine and stool achieved the highest score, 4.8. However, functions such as fingerprinting, facial recognition, and radio-frequency identification analysis garnered an average score of 3.9 and were deemed the least necessary according to the aggregate responses from all questionnaires. These results underscore the need for rigorous research and development of fecal care functionality to fulfill clinical requirements.

**Table 2:** Preferences for product features.

Variables and categories		Score, mean (SD)	Likert scale, n (%)				
			1	2	3	4	5
**Product features**							
	Automatic flush (remove all eliminations)	4.7 (0.59)	0 (0)	1 (1)	2 (3)	12 (18)	52 (78)
	Self-cleaning system	4.6 (0.70)	0 (0)	1 (1)	5 (7)	16 (24)	45 (67)
	Air drying function	4.4 (0.78)	0 (0)	2 (3)	6 (9)	19 (28)	40 (60)
	Self-cleaning (disinfection) of inner part of the device	4.6 (0.59)	0 (0)	0 (0)	4 (6)	16 (24)	47 (70)
**EMR^a^-integrated automatic analysis function**							
	Automated, accurate urine and stool output check capabilities	4.8 (0.48)	0 (0)	0 (0)	2 (3)	11 (16)	54 (81)
	Urinalysis (using urine test strip)	4.1 (0.98)	0 (0)	6 (9)	11 (16)	20 (30)	30 (45)
	Ability to record and classify bowels (by BSFS^b^) through algorithms	4.4 (0.69)	0 (0)	0 (0)	8 (12)	24 (36)	35 (52)
	User identification via fingerprint, face recognition, or RFID^c^	3.9 (1.05)	2 (3)	4 (6)	19 (28)	19 (28)	23 (34)

^a^EMR; electronic medical record.

^b^BSFS: Bristol Stool Form Scale.

^c^RFID: radio-frequency identification.

### Qualitative Results

#### Overview

The qualitative study identified four key themes associated with the Smart Excretion Care System: (1) reduction in workload, (2) patient indications, (3) improvement of automatic functions, and (4) prevention of nosocomial infections. Nurses emphasized the potential of the system to decrease their workload and increase efficiency, particularly for patients in the ICU and certain specialty wards. Suggestions for improvements focused on adding more automatic functions, including dermatitis prevention cream application, and adapting the device for different patient positions. The participants also highlighted the importance of daily cleaning to prevent nosocomial infections, albeit while acknowledging the limitations for immunodeficient patients and those with skin damage.

#### Theme 1: Reduced Workload

The interviewees highlighted the significant burden that EC places on nursing staff, with frequent diaper changes for patients with severe diarrhea being particularly time-consuming and physically demanding. This was viewed as one of the most challenging aspects of nursing, with additional strain due to the physical exertion required when dealing with heavier patients. The nurses expressed the hope that the use of the Smart Excretion Care System would increase work efficiency, save time, and reduce workload while also preventing skin diseases, UTIs, and diaper dermatitis in the patients. However, they also noted potential drawbacks, including the device’s large size, the need for repeated instructions, and the possibility of resistance from nurses due to perceived additional work.

I have to change diapers multiple times a day if the patient has severe diarrhea.

Participant #2

I am sure that changing patients’ positions and providing patients’ excretion care are among the top 3 hardest aspects of a nurse’s role regardless of the severity of the disease.

Participant #5

There happens to be a lot of heavy work involved in excretion care, and I suffer from back and wrist pain when the patients weigh more.

Participant #1

Common expectations among nurses regarding the benefits of using the device in a hospital setting included increased work efficiency, time savings, and workload reduction (Table S5 of Multimedia Appendix 1). From the patients’ perspective, it was anticipated that the device could aid in preventing skin diseases and UTIs, reducing the occurrence of diaper dermatitis, and alleviating embarrassment. However, several potential drawbacks were noted, including difficulty securing room space owing to the large size of the device, the need for repeated instructions to guardians or the users, and the possibility of nurses refusing to use it because of the additional work involved.

I think it will be very helpful not only to prevent dermatitis, UTIs, and other diseases but also to reduce the delay in my work due to excretion care.

Participant #10

I would like to use it because I can focus more on other tasks if the device can handle excretion work.

Participant #42

#### Theme 2: Patient Indications

The interviewees suggested that the device would be most beneficial for use in the ICU, followed by the neurosurgery/neurology and geriatric internal medicine wards (Table S2 of Multimedia Appendix 1). It was agreed that stroke, dementia, pneumonia, and postsurgical patients would benefit the most from this device. The patients’ level of consciousness and ability to express themselves were also identified as important considerations when deciding to use the device.

I think it should be first applied to patients who are conscious but have difficulty moving.

Participant #4

It would be great for unconscious patients.

Participant #7

I also agree that it should be first supplied in ICUs and then in comprehensive nursing care wards. If I understand correctly, the purpose of using this device is to reduce the workload of nurses, and assisting patients with elimination is one of the main roles of nurses working in comprehensive nursing care. Therefore, I think it would be better if it were initially distributed to ICUs and comprehensive nursing care wards.

Participant #31

#### Theme 3: Improvement of Automatic Functions

The interviewees suggested several areas for improvement in the Smart Excretion Care System (Table S3 of Multimedia Appendix 1). These included the addition of a function to automatically apply a dermatitis prevention cream, voice recognition, an alarm system for unusual excretion or device disconnection, remote controls for additional wash-and-dry features, disposable components, massage functions, and an automated panel displaying the device’s wearing time, amount of urine, and frequency of use. It was also suggested that the device be usable in the semi-Fowler position and made smaller to accommodate rooms with multiple patients.

It would be very comfortable to have an automatic cream spray-like function that helped in preventing pressure ulcers, diaper dermatitis, or any other skin diseases in patients.

Participant #22

To monitor the amount of elimination per hour is more important than the number of times.

Participant #2

It should also be usable when the patient is in the semi-Fowler’s position.

Participant #28

#### Theme 4: Nosocomial Infection

Regarding the device’s overall applicability, the participants discussed measures to prevent nosocomial infections (Table S4 of Multimedia Appendix 1). They recommended cleaning the device daily, including its waste cabin, water tank, and other components like the cup and hose. However, they also acknowledged that the device might not be suitable for patients with immunodeficiency and skin damage, given the requirement for continuous attachment.

I’m a little bit worried that there could be more contact infections, secretion infections, or bodily fluid infections…[O]f course you can sterilize the silicone and boil it…but you can’t boil the plastic.

Participant #45

It would be nice to be able to make a disposable bag and throw it away, which would reduce the risk of infection.

Participant #8

#### Theme 5: Willingness to Use the Smart Excretion Care System

In addition to the previously discussed themes, our thematic analysis examined nurses’ willingness to use and the expected benefit of the proposed Smart Excretion Care System in their daily routines (Table S5 of Multimedia Appendix 1). This aspect is crucial because, while understanding the perceived benefits and challenges is essential, it is also vital to determine whether nurses would be genuinely open to integrating this technology into their care practices. The findings revealed that while nurses acknowledged the potential advantages of the system, their willingness to use it depended on factors such as ease of use, reliability, training provided, and perceived patient comfort and dignity.

If the system proves to be reliable and doesn’t add extra tasks to our routine, I’d be willing to use it.

Participant #47

The training and support around the system will play a significant role in whether I’d use it daily.

Participant #30

## Discussion

### Principal Findings

This study aimed to identify the anticipated benefits and barriers to the development of the Smart Excretion Care System for clinical facilities. Focus group interviews were conducted to assess the burden of EC for nurses, the adaptation of the Smart Excretion Care System, the functions they wished to add, and the evaluation of pilot devices.

Building on the existing literature, our study underscores the critical gap in addressing the usability of Smart Excretion Care Systems. Previous research has predominantly focused on various domains of nursing care, such as safety, nutrition, counseling, and education [5-7]. However, despite the evident significance of EC, particularly in the context of an aging population and the associated challenges [1-4], studies focusing on the practical application and usability of smart systems for EC remain conspicuously absent. Most prior studies have either conducted exploratory investigations or worked toward standardizing nursing practices [8,9]. The development of numerous smart devices, such as smart mattresses and patient transport robots [17-23], is a progressive step. However, when it comes to EC, the emphasis has mainly been on biological signal collection and fecal analysis [24,25]. Therefore, our study is a pioneering endeavor to evaluate the usability of a comprehensive Smart Excretion Care System aimed at holistically addressing patients’ urinary and bowel elimination needs.

The focus group interview results revealed that the burden of EC was well understood. According to this study, the percentage of EC work during working hours and the number of delays in other work due to EC were highest in CNCWs. The biggest problems related to EC were irregular excretion and diarrhea, with 94% of patients needing fecal care in the highest ICU. This result may be because nurses in CNCWs are fully in charge of EC, like those in ICUs, but there are more patients per nurse in these wards than in ICUs. Regarding the burden on nurses, a previous study on intensive care nurses found the highest incidence of musculoskeletal pain was due to nursing tasks [34]. Other studies have analyzed the prevalence of musculoskeletal disorders and the physical burden on nurses, including urinary and bowel elimination care, position changes, and transfers, but there have been no studies on the burden of urinary and bowel EC [11-13].

The Smart Excretion Care System was investigated in terms of department, disease, and patients’ physical characteristics. There was a common opinion that ICUs are most in need of the Smart Excretion Care System. In terms of patient indicators, those with stroke or dementia were seen as most in need of such a system. Regarding patients’ physical characteristics, those who were unconscious or conscious but unable to move were thought to require EC the most. The findings here show that the burden of EC is related more to the physical characteristics of the patient than to the severity of the patient’s disease condition.

To define the method of disinfection or cleaning of the device, a group interview with the infection control team was conducted. The results suggested that the disinfection method should differ depending on the concerned part, such as the part of the cup, the hose, the main body, or the bucket. The consensus was that the cup part should be cleaned with a disinfectant and replaced when applied to other patients; the hose should be used for disinfection of the existing product itself; and the waste basket should be deposited with disinfectant. Immunocompromised patients and those with acupuncture wounds could be at a high risk of infection; therefore, medical staff should use their judgment when using the device. C. difficile infections induce apoptosis, so the device cannot be used even at a low level of disinfection. However, it would be available for patients with hepatitis A and polycystic-resistant bacteria, such as Methicillin-resistant Staphylococcus aureus and vancomycin-resistant Enterococci [35-37].

With regard to the possible improvements, most study participants wanted a notification sign that would be visible whenever the device detected abnormalities in the excrement. Size-related requirements were also important so that the machine could be placed at the foot of the patient’s bed. The participants hoped that a function would be developed to automatically spray creams or ointments to prevent pressure ulcers and IAD. In addition, the development of functions to measure the amount of excrement, such as hourly urine output, was seen as more important than measuring its frequency.

This study found that the position of the patients should be considered when using the Smart Excretion Care System. The proposed system can be used by patients in the supine position. However, almost all the participants said that the machine should be available to patients in the semi-Fowler position. These findings are in line with the results of prior studies suggesting that the semi-Fowler position is effective in several disease conditions [38-40]. This indicates the importance of developing the Smart Excretion Care System for use in the semi-Fowler position.

Seamless integration with EMRs is paramount to the broader landscape of health care informatics. This study evaluates the capabilities of the proposed Smart Excretion Care System in this context. The high ratings for automated urine and stool output checks, combined with the system’s ability to perform urinalysis and bowel classification, suggest the promising potential of this technology. This integration would not only enhance patient care by offering real-time, accurate data but also help health care professionals in making informed decisions, reducing manual entry errors, and improving the overall workflow. The slightly lower rating for user identification may indicate concerns regarding privacy and data security, underscoring the importance of robust encryption and security protocols in future iterations of the system.

### Limitations

This study has several limitations. First, the data were derived from a single tertiary hospital. For a more comprehensive understanding and generalization of our findings, future research involving multiple centers will be invaluable. Second, the qualitative research process, especially with semistructured interviews, carries inherent biases. While we did not explicitly introduce assumptions, the framing and phrasing of the questions, especially after prediscussions with nurses, might have led the participants in certain directions or limited the scope of their responses. Questions like “What are the expected benefits of using the Smart Excretion Care System device?” can inadvertently emphasize the positive aspects, whereas other questions may emphasize the challenges. Despite this, it is important to note that the questions were crafted with the genuine aim of understanding nurses’ perspectives on a novel medical device without any predetermined agenda. Third, although every effort was made to maintain objectivity, the interpretation of qualitative data was inevitably influenced by the researchers’ personal perspectives. To mitigate this, we adhered to the COREQ guidelines. We also cross-validated the deduced themes with multiple researchers to curtail potential biases.

Fourth, the nursing environments surrounding EC can differ significantly across hospitals and countries. Since our study provides insights from a specific setting, these findings may not be universally applicable. That said, the core challenges related to EC are likely consistent across many settings, offering valuable lessons for researchers and professionals elsewhere. Fifth, the metrics provided by the nurses, such as the percentage of EC work per shift, delays due to EC, and the estimated time for EC, were based on nurses’ perceptions rather than on precise measurements using scientific tools or time-motion studies. Future research should aim to quantify these aspects more accurately, possibly through detailed time-motion studies or analyses of nursing records. Sixth, a significant limitation is the study’s reliance on participants’ perceptions of a theoretical design without the experience of the actual functioning of the technology. This approach inherently required the participants to visualize and understand the proposed technology based on descriptions and potential demonstrations. Consequently, their perspectives may have been limited or even flawed because they did not have hands-on experience with the system in a real-world setting. Engaging participants in discussions about conceptual design may not capture the intricacies, challenges, and benefits they might encounter when using the technology in their daily routines. In future studies, beta testing the technology in a qualitative format could offer deeper and more accurate insights.

### Conclusion

This was a formative research study using focus group interviews to collect basic data for the development of the Smart Excretion Care System for clinical and community care settings. The required improvements to the pilot devices are meaningful for identifying the expected benefits, barriers, and consequent demands of the hospital’s Smart Excretion Care System and the burden of nurses’ EC. Furthermore, while our focus was primarily on alleviating the burden on nurses, we recognize the intrinsic value of the patient’s perspective, especially given the potentially invasive nature of this technology. Their voices and comfort are paramount because the aim of any health care technology is to enhance patient care. In future studies, it would be beneficial to incorporate patient feedback to ensure that the developed system is both efficient for caregivers and respectful of the patients’ dignity and privacy.

## Appendix

Multimedia Appendix 1 Supplementary tables.

## Abbreviations

CNCW: comprehensive nursing care ward
COREQ: Consolidated Criteria for Reporting Qualitative Research
EC: excretion care
EMR: electronic medical record
GW: general ward
IAD: incontinence-associated dermatitis
ICU: intensive care unit
IoT: Internet of Things
SNUBH: Seoul National University Bundang Hospital
UTI: urinary tract infection
