# Supplementary material for: Use of the Smart Excretion Care System Linked to Electronic Medical Records to Alleviate Nursing Burden and Enhance Patient Convenience: Mixed Methods Study
Source: JMIR Form Res. 2023 Oct 30;7:e36324. doi: 10.2196/36324 (PMC10644194; doi:10.2196/36324)
Supplement: Multimedia Appendix 1 [file formative_v7i1e36324_app1.docx]

Table S1. List of interview questions for participants

| **Theme** | **Questions** | |  |
| --- | --- | --- | --- |
| **Assisting patients with elimination** | 1. What is the percentage of excretion care work per shift? | |  |
|  | 2. How many times do other tasks get delayed due to assisting patients with elimination? | |  |
|  | 3. How long does it take to assist in excretion care once when using (min) | |  |
|  | a. bedpan | |  |
|  | b. diaper | |  |
|  | 4. What are the biggest factors that put a burden on your work when caring for patients who need excretion related nursing care (e.g., Bowel irregularities such as diarrhea, incontinence, constipation) | |  |
|  | 1^st^ | |  |
|  | 2^nd^ | |  |
|  | 3^rd^ | |  |
|  | 5. What percentage of the patients in charge require excretion related nursing care? | |  |
| **Indication** | 1. Please list the applicable indications that can be applied when classifying by | |  |
|  | a. departments (wards) | 1^st^ |  |
|  |  | 2^nd^ |  |
|  |  | 3^rd^ |  |
|  | b. diseases | 1^st^ |  |
|  |  | 2^nd^ |  |
|  |  | 3^rd^ |  |
|  | c. patient's physical characteristics | 1^st^ |  |
|  |  | 2^nd^ |  |
|  |  | 3^rd^ |  |
| **Needs** | 1. What features/functions should be added/improved for | |  |
|  | a. nurses | |  |
|  | b. patients | |  |
|  | c. clinical facilities | |  |
|  | 2. What do you think about the device’s size and appearance? | |  |
|  | 3. Where do you think the device should be placed? | |  |
| **Evaluation** | 1. What are the expected benefits of using a Smart Excretion Care System device' from the perspective of: | |  |
|  | a. nurses | |  |
|  | b. patients | |  |
|  | c. clinical facilities | |  |
|  | 2. What are the expected disadvantages of using a ' Smart Excretion Care System '? | |  |
|  | 3. What are the expected side effects in case of using a ‘Smart Excretion Care System’? | |  |
|  | 4. Are you willing to use it? If yes, why or if not, why not? | |  |

Table S2. Applicable indications of Smart Excretion Care System

| Theme : Applicable indications | | | |
| --- | --- | --- | --- |
|  | Intensive Care Unit | General Wards | Comprehensive Nursing Service Wards |
| 1. Please list the applicable indications that can be applied when classifying by type of departments | | | |
| 1^st^ | Intensive Care Unit | Intensive Care Unit | Intensive Care Unit |
| 2^nd^ | Neurosurgery / Neurology | Rehabilitation Medicine | Comprehensive Nursing Service |
| 3^rd^ | Geriatric Internal Medicine | Neurosurgery/Neurology | Neurosurgery / Neurology |
|  |  |  |  |
| 2. Please list the applicable indications that can be applied when classifying by diseases | | | |
| 1^st^ | Stroke | Stroke | Stroke |
| 2^nd^ | Dementia | Dementia | Pneumonia |
| 3^rd^ | Pneumonia | Obstetrics & Gynecology related disease | Surgery |
|  |  |  |  |
| 3. Please list the applicable indications that can be applied when classifying by patient's physical characteristics | | | |
| 1^st^ | Presence of consciousness | Presence of consciousness | Presence of consciousness |
| 2^nd^ | Ability to express one's opinion | Ability to express one's opinion | Ability to express one's opinion |
| 3^rd^ | - | Excretory system status | Fecal incontinence, at high risk from slip down |

Table S3. Requirements for improvement

| Theme: Requirements for improvement | | |
| --- | --- | --- |
| Intensive Care Unit | General wards | Comprehensive Nursing service wards |
| 1. What features/ functions should be added/improved for nurses, patients, and facilities? | | |
| For nurses  - Application/spraying of skin care cream (to prevent diaper dermatitis & pressure ulcers)  - Display of wearing time  For patients  - Reducing discomfort while wearing the device  - Additional cleaning and drying function  - Use of disposable motion control wrap  For facilities  - Decreasing cross infection rate by using disposable diapers  - Saving budgets | For nurses  - Measurement of amount, frequency and duration of urination  - Triggering of alarm when stool shape changes are observed  - Voice recognition  - Comprehensive monitoring dashboard  - Application/spraying of skin care cream (to prevent diaper dermatitis & pressure ulcers)  For patients  - Remote controller  For facilities  - Reduce waste  - Reduce expense | For nurses  - Simple Danuria mode  - Collection of specimen (elimination)  - Application/spraying of skin care cream (to prevent diaper dermatitis & pressure ulcers)  - Triggering of alarm when there is a gap between the user and device  For patients  - Usable in sitting position  - Massage function (hip or sacrum area)  - Triggering of alarm when leaking  For facilities  - Emptying water tank & waste cabin by using hose |
| 2. What do you think about the size and appearance of the device? | | |
| Compact size  (various size) | Compact size  (various size) | Compact size  (long length, short width) |
| 3. Where do you think the device should be placed? | | |
| Bedside (foot side) | Bedside (foot side) | Bedside (foot side) |

Table S4. Requirements of Infection Control Team

| Theme: Requirements for preventing nosocomial infection | | | |
| --- | --- | --- | --- |
| Waste container | Water Tank | Cup | Hose |
| 1. What are the requirements of the infection control team? | | | |
| *Cleansing period*  Once a day; anytime contamination is detected  *Order of interior cleansing*  Discard the waste →  Initial cleaning (remove left over clogs ) →  Disinfect (use liquid chemicals)  *Order of exterior cleansing*  Recommendation to use tissue-type disinfectants | *Cleansing period*  Once a day; anytime contamination is detected  *Order of interior cleansing*  Empty water tank →  Rinse the tank under running water (refill)  *Order of exterior cleansing* | *Cleansing period*  Once a day; anytime contamination is detected  *Order of interior cleansing*  Sink in water diluted with liquid chemicals  *Order of exterior cleansing*  - Recommendation to use tissue-type disinfectant  - Wash and disinfect separately (detach from the hose) before applying to other users | *Cleansing period*  Once a day; anytime contamination is detected  *Order of interior cleansing*  Disinfect the hose  *Order of exterior cleansing*  - Recommendation to use tissue-type disinfectants  - Recommendation to change before applying to other users. |
| Common requirements  - Recommendation to change before applying to other users.  - Be able to disassemble into small pieces to clean and disinfect the interior/exterior surface of the device | | | |
| 2. What do you expect in the Smart Excretion Care System from the perspective of the infection control team? | | | |
| - Necessary to develop a manual that allows for the disinfection of the entire waste container and water tank. (Consider that disinfection is insufficient if only 1/6 of the waste container is filled) | - Recommend to clean/disinfect at least once/day  - UV lamp seems unnecessary even though it is proven to be effective in some airborne infectious diseases such as tuberculosis  - Seniziam (Benzalkonium chloride) specified in the current | - Be able to break into many different parts to clean & disinfect | - Necessary to develop a manual that allows disinfecting the entire interior part of the hose. (Consider that disinfection is insufficient if only partial part of the hose filled with water mixed with liquid chemicals) |
| 3. What are your concerns about the Smart Excretion Care System? | | | |
| General concerns  - Not applicable to the patients with spore forming bacteria  - Able to apply to the patients with multidrug-resistant bacteria/Hepatitis A, such as MRSA and VRE. | | | |
| Difficult to handle approx. 10 liters of water | - Bacteria growth inside the water tank | Not available | Not available |
| MRSA; Methicillin-resistant Staphylococcus Aureus, VRE; Vancomycin-resistant Enterococcus | | | |

Table S5. Expected benefits.

| Theme: Expected benefits of the Smart Excretion Care System | | |
| --- | --- | --- |
| Intensive Care Unit | General wards | Comprehensive Nursing service wards |
| 1. What are the expected benefits of using the Smart Excretion Care System from a different perspective listed below? | | |
| For nurses  - Reduction in workload  For patients  - Time saving  - Reduction in the burden of work  - Increase in work efficiency  For facilities  - Reduction in the rate of UTI & pressure ulcer development  - Increase in the nursing personnel’s operational efficiency | For nurses  - Time saving  - Reduction in the burden of work  - Increase in work efficiency  For patients  - Overcoming embarrassment  - Reduction in the rate of diaper dermatitis & pressure ulcer development  For facilities  - Reduction in the rate of UTI & pressure ulcer development  - Increase in the nursing personnel’s operational efficiency | For nurses  - Increase in work efficiency (may focus on other duties)  For patients  - Overcoming embarrassment  - Reduction in the rate of diaper dermatitis & pressure ulcer development  For facilities  - Reduction in the rate of UTI & pressure ulcer development  - Increase in the nursing personnel’s operational efficiency |
| 2. What are the expected disadvantages of using Smart Excretion Care System? | | |
| - Massive size (relative to patients' ward size)  - Noisy | - Difficulty in explaining the device’s use to the patients and guardian repeatedly  - Restricted movement  - High cost  - Not applicable to patients who already suffer from ulcers | - Repeated attachment and detachment of the device |
| 3. What are the expected side effects of using the Smart Excretion Care System? | | |
| - possibility of pressure ulcer caused by the device  - limitations in changing position | - High cost  - Restricted movement | - Cross infection  - Possibility of pressure ulcer caused by the device |
| UTI; Urinary Tract Infection | | |

Consolidated criteria for reporting qualitative studies (COREQ): 32-item checklist.

| **No. Item** | **Guide questions/description** | **Reported on Page #** |
| --- | --- | --- |
| **Domain 1: Research team and reﬂexivity** |  |  |
| *Personal Characteristics* |  |  |
| 1. Inter viewer/facilitator | Which author/s conducted the interview or focus group? | Methods #11 |
| 2. Credentials | What were the researcher’s credentials? E.g. PhD, MD | Methods #11 |
| 3. Occupation | What was their occupation at the time of the study? | Methods #11 |
| 4. Gender | Was the researcher male or female? | Methods #11 |
| 5. Experience and training | What experience or training did the researcher have? | N/A |
| *Relationship with participants* |  |  |
| 6. Relationship established | Was a relationship established prior to study commencement? | N/A |
| 7. Participant knowledge of the interviewer | What did the participants know about the researcher? e.g. personal goals, reasons for doing the research | N/A |
| 8. Interviewer characteristics | What characteristics were reported about the inter viewer/facilitator? e.g. Bias, assumptions, reasons and interests in the research topic | N/A |
| **Domain 2: study design** |  |  |
| *Theoretical framework* |  |  |
| 9. Methodological orientation and Theory | What methodological orientation was stated to underpin the study? e.g. grounded theory, discourse analysis, ethnography, phenomenology, content analysis | Methods #5 |
| *Participant selection* |  |  |
| 10. Sampling | How were participants selected? e.g. purposive, convenience, consecutive, snowball | Methods #9 |
| 11. Method of approach | How were participants approached? e.g. face-to-face, telephone, mail, email | Methods #11 |
| 12. Sample size | How many participants were in the study? | Results #12 |
| 13. Non-participation | How many people refused to participate or dropped out? Reasons? | N/A |
| *Setting* |  |  |
| 14. Setting of data collection | Where was the data collected? e.g. home, clinic, workplace | Methods #11 |
| 15. Presence of non-participants | Was anyone else present besides the participants and researchers? | N/A |
| 16. Description of sample | What are the important characteristics of the sample? e.g. demographic data, date | Results #12 |
| *Data collection* |  |  |
| 17. Interview guide | Were questions, prompts, guides provided by the authors? Was it pilot tested? | N/A |
| 18. Repeat interviews | Were repeat inter views carried out? If yes, how many? | N/A |
| 19. Audio/visual recording | Did the research use audio or visual recording to collect the data? | Methods #11 |
| 20. Field notes | Were ﬁeld notes made during and/or after the inter view or focus group? | N/A |
| 21. Duration | What was the duration of the inter views or focus group? | N/A |
| 22. Data saturation | Was data saturation discussed? | N/A |
| 23. Transcripts returned | Were transcripts returned to participants for comment and/or correction? | N/A |
| **Domain 3: analysis and ﬁndings** |  |  |
| *Data analysis* |  |  |
| 24. Number of data coders | How many data coders coded the data? | Methods #11 |
| 25. Description of the coding tree | Did authors provide a description of the coding tree? | N/A |
| 26. Derivation of themes | Were themes identiﬁed in advance or derived from the data? | Methods #11 |
| 27. Software | What software, if applicable, was used to manage the data? | N/A |
| 28. Participant checking | Did participants provide feedback on the ﬁndings? | N/A |
| *Reporting* |  |  |
| 29. Quotations presented | Were participant quotations presented to illustrate the themes/ﬁndings? Was each quotation identiﬁed? e.g. participant number | Results #13-19 |
| 30. Data and ﬁndings consistent | Was there consistency between the data presented and the ﬁndings? | Discussion #26-28 |
| 31. Clarity of major themes | Were major themes clearly presented in the ﬁndings? | Results #14-23 |
| 32. Clarity of minor themes | Is there a description of diverse cases or discussion of minor themes? | Results #13-26 |
